# Supplementary material for: Acceptance and compliance with micronutrient powder (MNP) among children aged 6–23 months in northern Nigeria
Source: PLOS Glob Public Health. 2022 Oct 17;2(10):e0000961. doi: 10.1371/journal.pgph.0000961 (PMC10022258; doi:10.1371/journal.pgph.0000961)
Supplement: S1 Dataset — (ZIP) [file pgph.0000961.s008.zip › S8_Dataset/Spot Checks.zip!/Spot Checks/13072015_A_GU_AS_2_SC.pdf]

SPOT CHECK RECORDING FORM

State: Adamawa LGA: ____Guyuk___ Community: _Gugu

Name of caregiver (female): Asamma Joseph

Name or caregiver (male): ………………………

Sex of index child: _Female___ Age of index child (in months): _6months__________

Spot Check Information

1. **Date of MNP distribution to household** (circle option below based on LGA name above)**:**

- **Kebbi:** BirninKebbi&Jega LGA: **June 16**
- **Kebbi:** Danko-wasagu: **June 17**
- **Adamawa:**Song, Ganye: **June 18**
- **Adamawa:**Guyuk **June 19**

2. **Date of Spot Check Observation: ____13/7/2015_____**

3. **Number of sachets remaining**(Ask the caregiver to show you and count the specific number of sachets remaining)**:**

47

4. **Number of sachets that should remainusing one MNP sachet per day** (Analyst only)**:**

37

5. **Number of sachets remaining / number of sachets should be remaining**(Analyst only)**:**

____47____ / ____37_____ = __127_ %

Probes

Question 1. You have**XX** MNP sachets remaining. Explain how easy or difficult it was to use the MNP product on a daily basis for your child 6 – 23 months? (Circle one answer)

Very Difficult **Difficult** **Easy** **Very Easy**

Question 2. Describe any specific challenges that you faced that made the MNP difficult to use on a daily basis?

There is no challenge at all , I do not face any form of difficulty on how to use this product on a daily basis.

Question 3. Describe specific aspects of the MNP product that you or your child enjoyed that made it easier to use on a daily basis?

What makes it easy for me to use this product on a daily basis is the fact that my young child does not reject the kunu I mix the product into. That is what makes the jobeasy otherwise it would have been very difficult to feed the baby daily with this product.

Question 4. What questions do you have about the MNP based on your experience using it?

- I would like to know if the product has an expiry date? Some sid the product may likely expire before we consume the whole packets.

Question 5. What specific recommendations do you have that we can do as a program to make the MNP easier to use on a daily basis?

I would like federal ministry of health to reduce the number of days required to use the product that is from 60 days to 40 days because we as mothers are already in farming/ planting season and using this product daily for 60 days may be quiet difficult. We may likely forget to make kunu in the morning and in the evening. When we return from the farm we may be very tired and can easily forget to use it. But when the days are reduced we will be able to cope.
